# Assessing the association of sustainable agriculture with rural household food security (considering ecological, economic, and social aspects)

**DOI:** 10.3389/fnut.2022.899427

**Published:** 2022-10-20

**Authors:** Salman Sahraei, Mohammad Reza Pakravan-Charvadeh, Saeed Gholamrezai, Mehdi Rahimian

**Affiliations:** Department of Agricultural Economics and Rural Development, Lorestan University, Khorramabad, Iran

**Keywords:** sustainable agriculture, food security, socio-economic factors, Iran, nutrition

## Abstract

Due to the importance of sustainability in the world, we follow the missing pieces of the puzzle of sustainable agriculture and food security as a huge gap in the literature. To achieve this goal, a survey was analyzed to assess the linkage between these two concepts in the rural areas in Lorestan province in Iran. The status of food security was assessed using US Household Food Security Survey Module (HFSSM). A standard questionnaire extracted from the related literature was applied to calculate sustainability indicators. The results showed that the status of food security among households with no children (70%) was better than those with one or more children (28%). According to the Tobit model, none of the sustainability dimensions had a significant association with food insecurity. The results support the fact that the assessment of agricultural sustainability at the microeconomic level, short-term period, and a small, specified location cannot lead to reliable results due to the similar behaviors of farmers in these areas. The low level of agricultural sustainability is due to the lack of macroeconomic policies in the region to promote and disseminate the principles of sustainability, lack of plans and actions to promote sustainability by stakeholders and policymakers, ignorance of the target community, as subsistence producers who fall into a deprivation trap. The results suggest that policymakers should use two short-term and long-term strategies to improve the level of agricultural sustainability and increase food security status.

## Introduction

The world's population is projected to reach 9.7 billion by 2050 (1), and as a result, by and large, food production is projected to increase by around 70% (2). In response, countries have already taken several collective initiatives to reduce the global food crisis, such as attention to the concept of food security (3, 4). According to the 1996 World Food Summit, “Food security exists when all people have physical and economic access at all times to adequate, safe and nutritious food that meets the nutritional needs and nutritional preferences for an active and healthy life”. High food insecurity and providing enough foods for all people has increased the work of policymakers and administrations globally to support more sustainable agriculture in the world (5). The first political operationalization at the United Nations Conference on the Environment and Development in 1992 was postulated: Everyone has the right to a decent life (11). The 2015 UN Agenda finally approached sustainable development based on ”ending needs“, such as poverty and hunger, and with the key phrases ”fulfilling human potentials in dignity and equality and in a healthy environment“ (6–8). The United Nations designed the SDGs targets as a collection of 17 interlinked global goals to be a ”blueprint for achieving a better and more sustainable future for all“. At the same time, feeding the more than 690 million hungry people today - and the other 2 billion people who will face hanger by 2050 - requires a profound change in the global food and agriculture system (9). Increasing agricultural productivity and sustainable food production is crucial to help reduce the risk of starvation. The two concepts of food security and sustainability have several features in common. They are broad and complex concepts used by various disciplines and non-governmental groups such as NGOs and governments, which often form their definitions (10, 11). Although all global institutions and policymakers emphasize sustainable agriculture as an appropriate instrument for providing the needed foods, an important question remains: Is there always a significant linkage between food security and sustainable agriculture? In other words, does sustainability lead to reducing food losses and enhancing food security status? We need a novel way to look at the linkage between these two inseparable concepts.

Few empirical studies in many countries have shown that sustainable agriculture is needed to improve food and nutrition security, and that the former can strengthen the latter. Chowdhury et al. (3) recommend any policy at the international, national, and local levels aimed at achieving food security should combine measures to address key global sustainability challenges (3). Skaf et al. (4) contend sustainable management of agricultural production provides access to healthy, wholesome, and nutritious food for the growing population (4). Another study suggests that sustainability should be considered as a part of the long-term time dimension in the assessment of food security (10). Another scientific group claims that the linkages between food sustainability and food and nutrition security intersect at global, national, local, and household levels (12). In fact, sustainable agriculture and sustainable food systems support food security (13). Nkomoki et al. (8) proved although the adoption of crop diversification and agroforestry as sustainability indicators is associated with higher household food security, other indicators, including intercropping and planting basins are not significantly associated with food security. Although theoretical studies emphasize that adhering to the principles of sustainable agriculture has a positive effect on food security, we face a dilemma: more production for food security and sustainable agricultural production. Some suggest that sustainable agriculture, at least in the short term and without appropriate support, may lead to a reduction in production, which in turn can reduce the motivation of farmers to implement sustainable practices.

As an important gap, none of these studies endeavored to demonstrate whether this nexus is always affordable in all areas or not. We think that a significant association between food security and sustainable agriculture is strongly linked to the geographical structure and farmers' behavior and varies region by region. The primary purpose of this paper is to find an appropriate answer to this puzzle with the inclusion of sustainability as an independent factor (in various models based on triple dimensions of sustainability) in defining and changing the position of food security. This article highlights the pros and cons of providing a deep and comprehensive understanding of the integration, links, and gaps between sustainable agriculture and food security in rural areas. Based on these explanations, the following hypotheses will be tested:

H1- More than half of farmers are food insecure in the study location.
H2- Most of the farmers are sustainable in the process of agricultural activity.
H3- Economic sustainability of farmers is positively associated with food security.
H4- The sustainable agriculture is positively and significantly associated with food security.

## Materials and methods

### Study area

**Figure 1:**
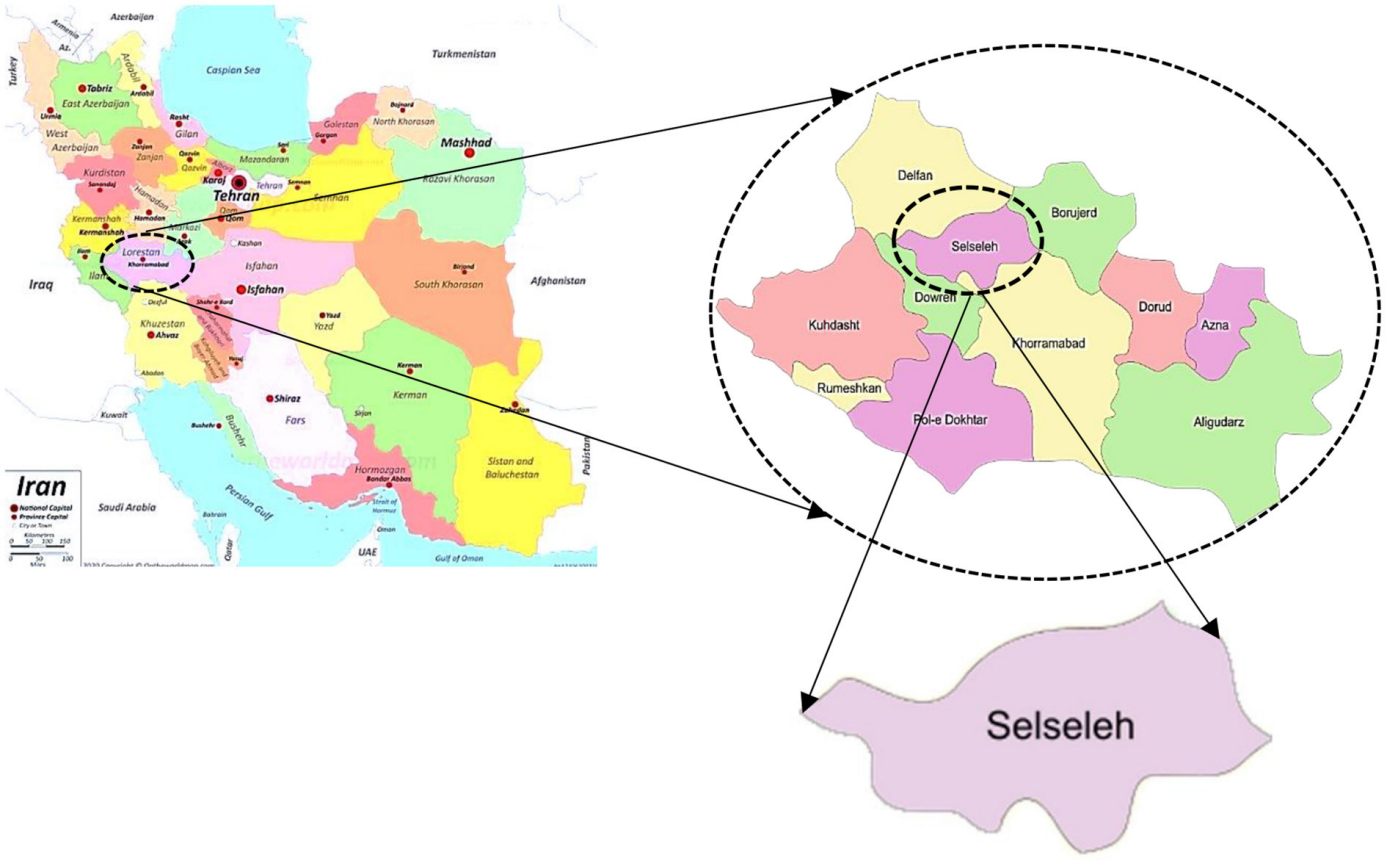
The map of study location.

The present study was carried out in Aleshtar city in Lorestan province as a province of the western Iran in the Zagros Mountains (Figure 1). This city is the center of Selseleh city in the north of Lorestan province. Information of Iran Statistics Center shows Aleshtar, with a population of 34,133 people in 2019, is ranked 238 among country's cities and ranked 8 among the cities of Lorestan province.

### Study sample and data collection process

The data were collected using a questionnaire in the study location. We randomly selected 140 farmers using the Cochran formula. The questionnaire included two sections. The first section consists of a food security questionnaire based on the U.S Household Food Security Survey Module, and the second section consists of questions which were extracted from the literature to calculate and indicator of agricultural sustainability on that farm. Prior to collecting the data, the validity of all questions of the second section were assessed by a team of experts consisting of an agricultural economist, agricultural extension expert, psychology, environment, and social science experts, and three nutritionists. To reduce the possibility of error in completing the questionnaires, a pilot study was conducted on 20 rural households. Before collecting the data, the interviewers, recruited from students of agricultural economics, were trained in a 2-day workshop to ensure coordination and to reduce interpersonal variation in the data collection process. The questionnaires were filled out through face-to-face interviews with breadwinners (farmers).

### Sustainability indicator

**Figure 2:**
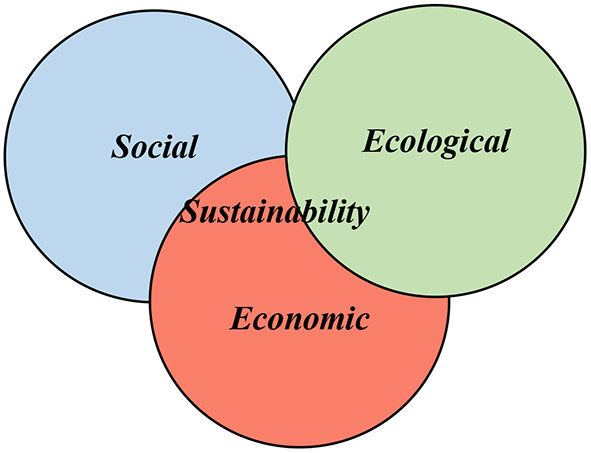
The triple bottom line.

To calculate sustainable agricultural indicator, a questionnaire was used based on the literature (10, 14–19). For this aim, all items were categorized into three sub-categories, including economic, social, and ecological aspects (Figure 2) (7). All related items were extracted from the related papers in the field of sustainability, and some questions were added to the item collection based on the researchers' knowledge of the study area (Table 1).

**Table 1:** Item description of different dimensions of agricultural sustainability.

**Dimension**	**Item description**	**Scale**
The economic dimension of agricultural sustainability	Non-agricultural income (per year)	Local currency
	Amount of loans received	Local currency
	Value of sold product	Local currency
	Harvesting cost with harvester and combine per hour	Local currency
	Electricity cost per hour	Local currency
	Cost of services (insurance, etc.) per year	Local currency
	Labor cost in the growing season	Local currency
	Fertilizer cost	Local currency
	Pesticide cost	Local currency
	Income from the sale of straw from one hectare	Local currency
The social dimension of agricultural sustainability	Access to agricultural inputs	Likert (1–5)
	Sales and marketing quality	Likert (1–5)
	Input availability	Likert (1–5)
	Wheat-related support services provided by stakeholders	Likert (1–5)
	Educational and extension services related to wheat provided by those involved	Likert (1–5)
	Participation in extension training programs	Likert (1–5)
	Cooperation with other farmers	Likert (1–5)
	Farmer's trust and credibility among the villagers	Likert (1–5)
	Contact and benefit from exemplary and leading farmers	Likert (1–5)
	The farmer's attitude toward sustainable cultivation of wheat	Likert (1–5)
	Level of farmer technical knowledge about wheat	Likert (1–5)
The ecology dimension of agricultural sustainability	Herbicide use	Liter/Ha
	Use of fungicides	Liter/Ha
	Insecticide use	Liter/Ha
	Phosphate fertilizer use	Kg/Ha
	Potash fertilizer use	Kg/Ha
	Nitrogen fertilizer application	Kg/Ha
	Use of animal manure	Kg/Ha
	Share of irrigated cultivation of the total area under cultivation	%
	Percentage of family employed in agriculture relative to total	%
	household members	%
	Farm size	Ha
	Number of farm plots	Number
	Use of agricultural machinery during the planting to harvest period	Hours
	Production yield	Ton/Ha
	Seed consumption	Kg/Ha
	Amount of irrigation water consumed	M2/Ha
	Number of irrigations	Number
	Number of light livestock	Number
	Number of heavy livestock	Number
	Percentage of hand harvest	%
	The area of rain-fed lands turned blue in the last 5 years	Ha
	Area of irrigated land converted to rain-fed in the last 5 years	Ha
	Increased area of agricultural land in the last 5 years	Ha
	Decrease in agricultural land area in the last 5 years	Ha
	Area of rangeland lands converted to agricultural lands in the last 5 years (hectares)	Ha
	Area of forest lands converted to agricultural lands in the last 5 years	Ha
	Rocky land area	Ha
	Level of uncultivated land in the last 5 years	Ha
	Rough land area	Ha
	Area of cultivated land without plowing to the area under crop	Ha
	Area of plowed land with pen or goose plow	Ha
	The ratio of sloping lands to total lands	Ha
	The ratio of sown fields with linear work	Ha
	The ratio of hand-sewn land	Ha
	Percentage of land whose straw has been burned	%
	Percentage of lands with concrete streams	%
	Percentage of land with covered streams	%
	Percentage of land covered by pressurized irrigation	%
	Area of cultivated land with a reversible plow	Ha
	Disc plowed area	Ha
	Area of land where several crops are cultivated (mixed cultivation)	Ha
	Multi-crop land area	Ha
	Land area covered by crop rotation	Ha
	Percentage of crop yield due to harvester harvesting	Ha
	Percentage of combine harvest without harvesting straw	%

As above-mentioned explanations, the validity of all questions was confirmed by an expert panel. The economic dimension of sustainable agriculture included ten items. All items of this criteria were alluded to all income and costs of farmers during the production process.

The social dimension consisted of 11 items to follow the farmers' knowledge and behavior, and their attitude toward sustainable agricultural activities. Finally, the ecological dimension which included 45 items was alluded to all interactions with agricultural activity, the environment, and environmental protections.

It was necessary to multiply each indicator by its weight before combining the indices. Other studies have used the principal component (PC) method to measure the weight of indicators in measuring stability (20–22). Hence, in the third step, the weight of each indicator was determined using this method. Then, the score for each indicator was multiplied by the identified weight. Next, the mean of sustainability was calculated in each dimension (Equations 1–3):


(1) $$\begin{array}{l} \text{Economic sustainability }\\ =\frac{\sum \left[\left(Eco1\times WEco1\right),...,\left(Eco10\times WEco10\right)\right]}{10} \end{array}$$

Where Eco_1_–Eco_7_ are scores given to 10 items of the economic indicator, and WEco _1_–WEco _10_: the weight of 10 items of the economic indicator in evaluation.


(2) $$\begin{array}{l} S\text{ocial sustainability}\\ =\frac{\sum \left[\left(Soc1\times WSoc1\right),...,\left(Soc11\times Wsoc11\right)\right]}{11} \end{array}$$

In which, Soc_1_–Soc_11_ are scores given to the social items of agricultural sustainability, and WSoc_1_–WSoc_11_ are the weight of the social items to calculate the social dimension of agricultural sustainability.


(3) $$\begin{array}{l} E\text{cological sustainability}\\ =\frac{\sum \left[\left(Ecol1\times WEcol1\right),...,\left(Ecol45\times WEcol45\right)\right]}{45} \end{array}$$

Where Ecol_1_ – Ecol_45_ are scores given to the ecological items to calculate the ecological dimension of agricultural sustainability, and WEcol_1−_ WEcol_45_ are the weight of the ecological items. By summing up the mean of three dimensions together, the total sustainability (CI) value of SA was calculated in each area as:


(4) $$\begin{array}{l} CI(\text{Total Sustainability})\;\\ =\sum (\text{Economic Sustainability},\text{ SocialSustainability},\\ \text{ Ecological Sustainability}) \end{array}$$

Through Equation (6), the level of SA for each farmer is obtained. CI has also been used by other researchers (23).

### Food security indicator

To assess food security status, the US Household Food Security Survey Module (HFSSM) was applied. The standard questionnaire in this method, which includes ten questions for adults and eight questions for children, is designed for a period of 1 year (12 months) or 1 month (with some modifications in questions 8, 12, and 14, and also in the number of occurrences) (24, 25) (Table 2). Using this approach, the household food security level is classified into four groups: high food security, marginal food security, low and very low food security. This status can be calculated for households with and without children. Also, this method can be used to assess the status of children's food security in three categories, including high or marginal food security, low and very low food security levels (25).

**Table 2:** Questions included in the food security scale.

**Item number**	**Item description**	**Scale**
2	Worried food would run out	0–2
3	Food bought didn't last	0–2
4	Couldn't afford to eat balanced meals	0–2
5	Relied on a few kinds of low-cost food for children	0–2
6	Couldn't feed the children a balanced meal	0–2
7	Children not eating enough	0–2
8	Adult cut size of meals or skipped meals	0–1
8a	Adult cut or skipped meals, three or more months	0–2
9	Respondent ate less than felt they should	0–1
10	Adult hungry but didn't eat	0–1
11	Respondent lost weight	0–1
12	Adult did not eat for whole day	0–1
12a	Adult did not eat for whole day, three or more months	0–2
13	Cut size of child's meals	0–2
14	Child skipped meal	0–1
14a	Child skipped meals, three or more months	0–2
15	Child hungry but couldn't afford more food	0–1
16	Child did not eat for whole day	0–1

Based on the corrections made in recent years, the sum of positive answers to the questions asked in the HFSSM questionnaire is considered as the score of each household and is the basis of the final classification. The types of food safety classification for households with and without children are as follows:

1- For households with at least one child under 18 years
  - Total positive responses = 0, high food security
  - Total positive responses = 1–2, marginal food security
  - Total positive responses = 3–7, low food security
  - Total of positive responses = 8–18, food security is very low
2- For families without children
  - Total positive responses = 0, high food security
  - Total positive responses = 1–2, marginal food security
  - Total positive responses = 3–5, low food security
  - Total positive responses = 6–10, food security is very low

High and marginal food security status of households are classified as food secure households, and low and very low food security status are classified as food insecure households (25).

### Quantitative model and variables description

The Tobit model is a regression model in which the observed variable range depends on some kind of censored levels. The term was invented by Arthur Goldberger, referring to James Tobin, who developed the model in 1958 to reduce the problem of zero inflation data to observe household spending on durable goods (26). The Tobit method can be easily extended to handle shortened samples and other non-randomly selected samples (27). The general form of the Tobit model is shown in the following relation (28):


$$\begin{array}{l} y_{i}=\gamma^{\prime }z_{i}+u_{i}\;i=1,2,\ldots ,N \end{array}$$

$$\begin{array}{l} y_{i}^{*}=\gamma^{\prime }z_{i}+u_{i}\;\text{if }y_{i}≻0 \end{array}$$

$$\begin{array}{l} y_{i}^{*}=0\;\text{if }y_{i}\leq 0 \end{array}$$

In which, y_i_ is an unobserved or latent variable, $y_{i}^{*}$is an observed variable, γ′ is a vector (1^*^k) of parameters that should be estimated, z_i_ is the vector of independent variables, u_i_ is the standard error of the equation that is independent of explanatory variables and assumes a normal distribution with a mean of zero, and fix variance of σ_*u*_ (28). In the Tobit model of the present study, 0 was considered for the households with no food insecurity status, and the calculated indicator of food insecurity using HFSSM was assigned to households who faced several levels of food insecurity (11).

Generally, Tobit is a regression model used for data with a discrete and continuous part. In different regression models, the analysis data are either discrete or continuous, but in the Tobit model, there is a pattern of combining both types of data. In other words, Tobit can be considered as an extension of the probit method and an appropriate approach to dealing with censored data. The coefficients are calculated using the maximum log-likelihood function, which indicates the consistency of the model (27).

To assess the goal of the study, some independent factors were added to the Tobit regression based on the literature review (2, 9, 29–34). These factors are the characteristics of the head of the household, the status of properties, the financial status of the households, and the information of the household's members. All these factors are shown in Table 3.

**Table 3:** The description of all used factors in the Tobit regression model.

**Factors**	**Scale**	**Description**
Sex of head of household	(0–1)	Male = 1; Female = 0
Married status of head	(0–1)	Married = 1; Single = 0
The status of the head's job	(0–1)	Employed = 1, Unemployed = 0
The status of the head's education	(1–7)	Illiterate = 1; rudimentary= 2; secondary school = 3; diploma = 4; associate degree = 5; bachelor = 6; master and higher = 7
The status of the mother's education	(1–7)	Illiterate = 1; rudimentary= 2; secondary school = 3; diploma = 4; associate degree = 5; bachelor = 6; master and higher = 7
The number of female children	Number	Number of female children within a household
The number of male children	Number	Number of male children within a household
The number of under six-aged children	Number	Number of under-six years old children within a household
The age of the household's head	Year	The age of head of household (breadwinner)
The age of the mother	Year	The age of mother of household
The number of the employed members	Number	Number of employed members within a household
Agricultural activity	Year	Years of agricultural activity of farmer
Homeownership status	(0–1)	Personal home = 1; Rental home = 0
The monthly rent	Rial (Local currency)	Monthly payment of household to rent a home
Mortgage	Rial (Local currency)	The fixed money which household pays to a landlord for renting a home
Home area	M^2^	The area of the household's home
The number of rooms	Number	Number
The age of home	Year	Year
Distance from city center	Km	Km
Personal saving	(0–1)	Having = 1; Not having = 0
Receiving a loan from a bank	(0–1)	Yes = 1; No = 0
Receiving governmental supports	(0–1)	Yes = 1; No = 0

## Result

**Figure 3:**
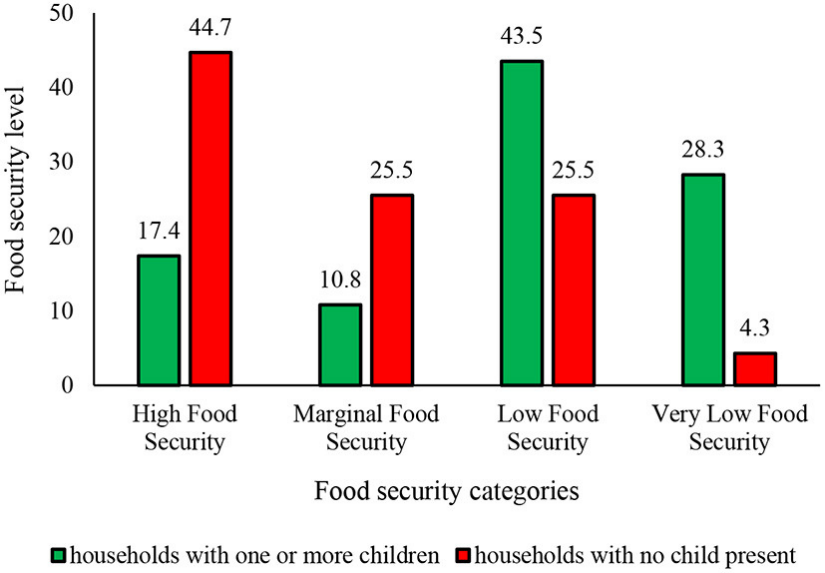
The status of household food security based on having and not-having child.

Assessing the food security status of the households showed that about 27% of those with one or more children are food secure (in high and marginal levels), while 73% of these households face various types of food insecurity including low and very low levels. On the other hand, 70% of the households with no child were food secure, while about 30% the households faced food insecurity (Figure 3).

According to the Table 4 the status of food security among households with no child was better than those with one or more children. The greater difference among households with and without children in terms of food security level is related to high and very low food security in the study location.

**Table 4:** The status of food security for households with and without child.

**Food security**	**Households with one**		**Households with no**	
**level**	**or more children**		**child present**	
	**Number**	**Percent**	**Number**	**Percent**
High food security	8	17.4	42	44.7
Marginal food security	5	10.8	24	25.5
Low food security	20	43.5	24	25.5
Very low food security	13	28.3	4	4.3
Food secure	13	28.2	66	70.2
Food insecure	33	71.8	28	29.8

Assessing the status of food security of children revealed that about 65% of them faced low food security, while only 32% of these children were food secure in two categories, including high and marginal levels (Table 5).

**Table 5:** The status of children's food security.

**Food security level**	**Number**	**Percent**
High or marginal food security	15	32.6
Low food security	30	65.2
Very low food security	1	2.2

Over half of the respondents (67.8%) were ecologically unsustainable, while about 32% of the total sample had ecological sustainability. Fifty-seven percentage of the farmers faced economic unsustainability, and 72% were socially unsustainable. Of the total sample, about 65% of the participated farmers faced total unsustainability. According to this result, most of the farmers were unsustainable in agricultural activity (Table 6).

**Table 6:** The status of agricultural sustainability of local farmer.

**Dimension**	**Sustainable**		**Not-sustainable**	
	**Number**	**Percent**	**Number**	**Percent**
Ecologic	45	32.2	95	67.8
Economic	59	42.2	81	57.8
Social	38	27.2	102	72.8
Total	48	34.3	92	65.7

The results of the sustainability status of the participating farmers in different categories of food security level are shown in Table 7. The high level of ecological and social sustainability is related to food secure group, while the highest level of the economic sustainability occurred in the food insecurity group with severe hunger. The score of the total sustainability indicator revealed the highest level was related to food secure and sever food insecure groups, respectively.

**Table 7:** The status of household's food security and agricultural sustainable dimensions in the study location.

**Food security Level**	**Number**	**Percent**	**Sustainability dimensions**			
			**Ecological**	**Economic**	**Social**	**Total**
Food secure	79	56.4	1.14	0.93	1.04	3.12
Food insecure without hunger/marginal	25	17.8	1.06	0.80	0.95	2.81
Food insecure with hunger/moderate	9	6.5	0.35	1.05	0.99	2.40
Food INSECURE with Hunger/severe	27	19.3	0.73	1.35	0.92	3.01

**Figure 4:**
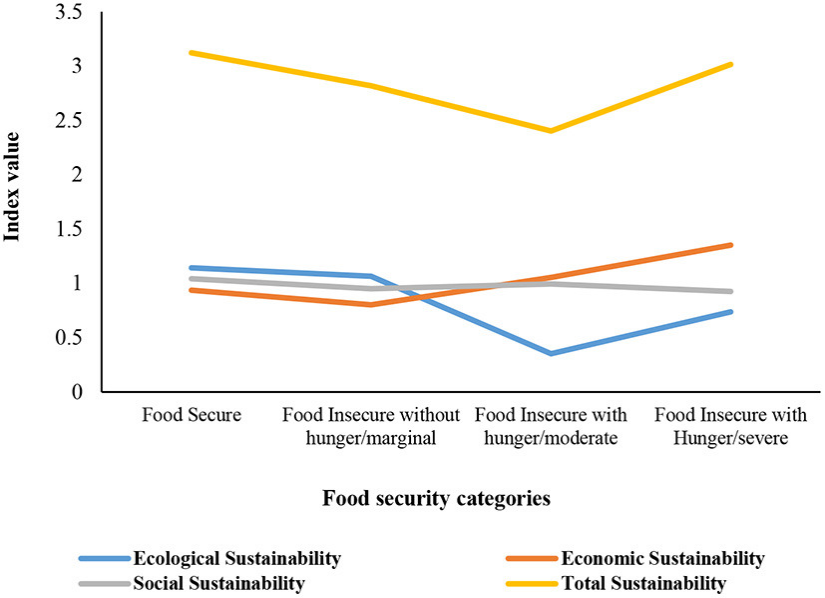
The status of ecological, economic, and social sustainability in different categories of food security.

Figure 4 demonstrates that the social dimension of sustainable agriculture is approximately fixed among all categories of food security level. Although most changes of sustainable indicators occurred in ecological and total indexes, these indicators were altered in an identified interval.

The results of the Tobit model are reported in Table 8. Four Tobit models were estimated in which each dimension of sustainability was added as an independent factor. Sex of head of household, head's job, agricultural activity, monthly rent, number of rooms, personal saving, and receiving a loan from a bank were significantly and inversely associated with food insecurity. The number of female and male children within a household, home area, age of home, distance from the city center, and receiving governmental support had a significant and direct association with food insecurity. The results demonstrated that none of the sustainability dimensions had a significant association with food insecurity.

**Table 8:** The results of censored regression (Tobit model) of households' food insecurity.

**Factors**	**Model (1)**	**Model (2)**	**Model (3)**	**Model (4)**
	**Considering**	**Considering**	**Considering**	**Considering**
	**ecology**	**economic**	**social**	**total**
Sex of head of household	−1.758**	−1.919**	−2.129***	−1.917*
Married status of head	2.648	2.748	2.738	2.757
The status of the head's job	−2.238**	−2.010**	−2.072	−2.151**
The status of the head's education	−0.220	−0.271	−0.234	−0.223
The status of the mother's education	0.372	0.315	0.333*	0.381
The number of female children	0.942***	1.013***	0.935**	0.937***
The number of male children	0.976***	1.010***	0.950***	0.965***
The number of under six-aged children	0.667	0.471	0.769	0.648
The age of household's head	−0.006**	−0.003**	−0.012**	−0.012***
The age of mother	0.010	0.001	0.007	0.010
The number of employed members	−1.053	−0.942*	−1.118**	−1.027
Agricultural activity	−0.059**	−0.059*	−0.058*	−0.056*
Homeownership status	2.188	1.697	0.760	2.241
The monthly rent	−6.41*10^−8**^	−7.19*10^−8**^	−1.46*10^−8^	−6.99*10^−8**^
Mortgage	2.18*10^−7***^	2.12*10^−7***^	1.47*10^−7^	2.23*10^−7***^
Home area	0.005**	0.005***	0.006***	0.005**
The number of rooms	−1.699*	−1.669*	−1.735	−1.737**
The age of home	0.095***	0.109***	0.090***	0.099***
Distance from city center	0.181***	0.203***	0.199***	0.180***
Personal saving	−4.205***	−3.915***	−4.297***	−4.148***
Receiving a loan from a bank	−1.156***	−0.992***	−1.236***	−1.209***
Receiving governmental supports	1.805***	1.733***	1.969***	1.817***
Ecology sustainability	−0.010			
Economic sustainability		0.499		
Social sustainability			2.488	
Total sustainability				0.087
Log pseudo-likelihood	−243.60	−243.03	−243.02	−243.49
The average of VIF	3.01	3.03	3.04	3.06

*, **, ***Significant at the level of 10%, 5%, and 1% respectively.

**Figure 5:**
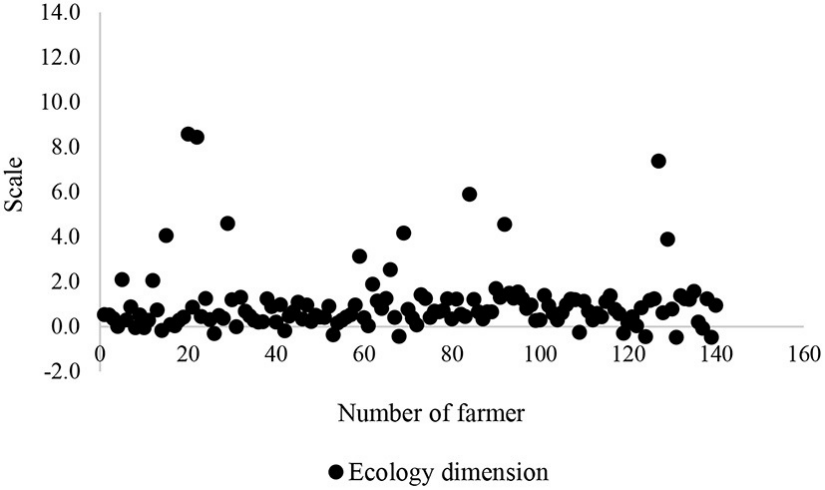
Scatter of the ecology dimension of the agricultural sustainability of local farmers.

**Figure 6:**
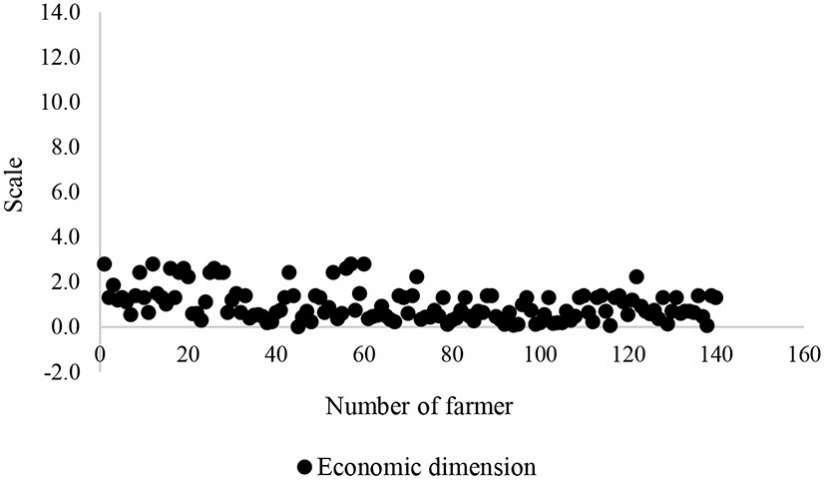
Scatter of the economic dimension of the agricultural sustainability of local farmers.

**Figure 7:**
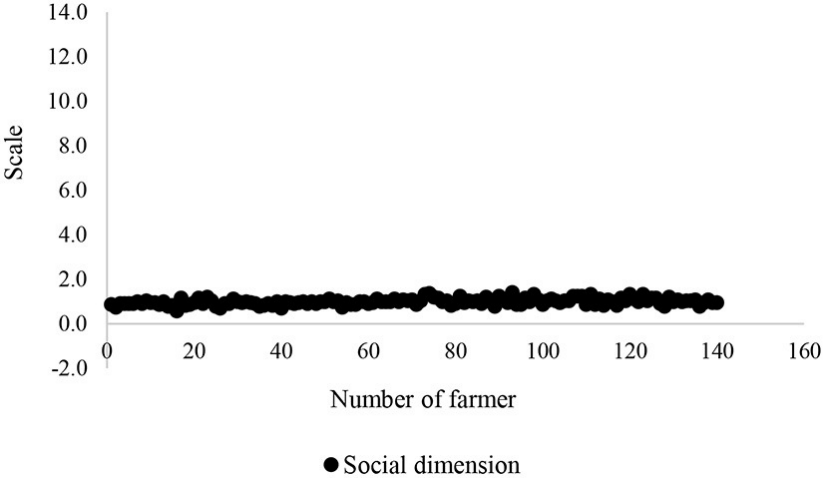
Scatter of the social dimension of the agricultural sustainability of local farmers.

**Figure 8:**
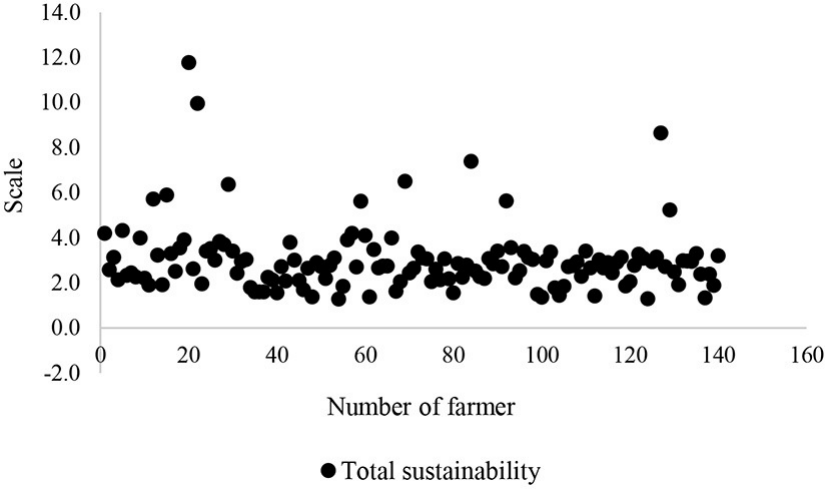
Scatter of the total score of the agricultural sustainability of local farmers.

To be confident in validity of an insignificant association of sustainability dimensions with food insecurity, the scatter plots of all these dimensions were depicted (Figures 5–8). All plots revealed that these dimensions are located in a specified range, and therefore, a high distribution is not viewed. These dimensions were altered in a specific interval for all farmers. Thus, there is no significant difference among the farmers in terms of agricultural sustainability behavior.

Among these dimensions, the behavior of the farmers is very close to each other in terms of social dimension.

## Discussion

The present study not only highlights the alarming rate of food insecurity for the households in this agricultural region, but also describes the association between food insecurity and sustainable agriculture among farmers and determines the associated factors. We recognized three hypotheses to organize the study.

H1- More than half of farmers are food insecure in the study location.

Assessment of household food security status showed that having and not having one or more children overshadows food security status. Food insecurity was more prevalent in households with one or more children than those without any children. Households who have at least a child have to allocate more income to prepare the needed foods for the members. Also, a part of the household's income should be paid for other costs instead of food consumption. This situation can affect food security. Some studies reported that having at least one child can affect a household's food security (33–37). About 28% of households who have at least one child faced very low food security, which shows the importance of the attention to these households in food aid programs by policymakers and governmental institutions (38, 39). About two-thirds of children of the participating households (about 67%) faced low and very low food security. The literature confirmed two groups, including women and children, have more vulnerability to food insecurity and should be supported by the related institutions (33, 40, 41).

H2- Most of the farmers are sustainable in the process of agricultural activity.

Of the total sample, 72.8% of the farmers were socially unsustainable. The check of participating households' responses showed that the knowledge of farmers about agricultural activities and cooperation of farmers with each other play a key role in controlling the social dimension of sustainable agriculture. The results revealed the low level of these dimensions in the study location. Some studies suggest that the low level of the social dimension of sustainability is the main reason for the low level of total sustainability (42–44). Despite the increasing recognition of the role of agriculture in the protection of the social heritage of territories, their traditions, and cultures, the assessment of the social dimension of sustainability has received less attention than the assessment of ecological and economic sustainability (14).

H3- Economic sustainability of farmers is positively associated with food security

The economic dimension of sustainable agriculture showed that the farmers cannot optimally allocate their financial resources to the cultivation process. The high level of service cost, input costs, and low level of efficiency can be introduced as the important reasons for 58% of the economic unsustainability of the participated farmers. The economic sustainability of agricultural activities is a fundamental restriction on the endurance of farm systems over time. In recent decades, this facet has become more vital, due to decreasing public support for agriculture and the intensification of global trade in agricultural productions. As a result, farmers face increasingly difficult decisions to improve the levels of competitiveness and profitability of traditional agricultural products (14).

About 67% of the total sample was ecologically unsustainable. The review of items of this dimension demonstrated that the high consumption of pesticide, insecticide, fertilizer, the inappropriate ways of cultivation, excessive water consumption, and land degradation are essential reasons for the low level of the ecological dimension of sustainable agriculture. Some scholars believe that ecological sustainability is the most important issue to provide healthier foods, and eventually, food security in a society (45). (46) contend that the ecological impacts on sustainable food consumption is inevitable, because it directly affects people's health (46).

H4- The sustainable agriculture is positively and significantly associated with food security

To find the answer to an important question of the present study, a Tobit model was estimated to figure out the association of sustainable agricultural dimensions with food security. The result showed that none of these dimensions were significantly associated with food security status in the study location. Although some scholars believe that there is a significant association between sustainable agriculture and food security in different regions in the world (4, 8, 47), and one can't exist without the other (10), this is strongly linked to the assessment period and the type of area. The results of the present study proved an insignificant association because most farmers were unsustainable and their behavior were close to each other. This similarity includes the economic, social, and ecological dimensions of sustainable agriculture. For this reason, a significant association between sustainability indicators (as independent factors) and food security status cannot be achieved. Also, as Nkomoki et al. (8) contended, the type of sustainability indicator can affect the results. The local farmers don't have a long-term vision and only want to catch instantaneous output without considering a sustainable production process.

## Conclusion

In the present study, we followed an important puzzle of the association of agricultural sustainability with food security using a quantitative approach. First, although households who have one or more children were more likely to be food insecure than those without any child, the status of food insecurity was dangerously high in the study location. Financial supports in the form of food subsidies, direct income payment, and food baskets are necessary to increase food security. Second, government and stakeholder engagement in promoting sustainable management programs can play an important role in raising awareness of sustainable agriculture activity, because this knowledge is a seriously lacking in the study location. The low level of agricultural sustainability is due to the lack of macroeconomic policies in the region to promote and disseminate the principles of sustainability, lack of plans and actions to promote sustainability by stakeholders and policymakers, ignorance of the target community, being subsistence, and fall into the deprivation trap. We suggest that policy makers use two short-term and long-term strategies to improve the level of agricultural sustainability. Short term, the development of educational programs for farmers, implementation of extension instructions, identifying specific policies, encouraging environmentally friendly farming methods, and strategies in the line of sustainability can contribute the farmers for being on the path of agricultural sustainability. As the long-term policies, land consolidation, mechanization of farms, production and supply of sustainable inputs, including organic fertilizers and organic pesticides, modification of cultivation pattern for development of water-less crops, and formation of agricultural cooperatives are suggested. Third, the results support the fact that the assessment of agricultural sustainability at microeconomic level, short-term period, and a small specified location cannot lead to reliable results due to the close behavior of farmers in these areas. Therefore, it cannot be strongly inferred that there is always a significant association between agricultural sustainability and food security in all regions. The results of this study can support farmers and policymakers responsible for ensuring sustainable management of agricultural production while providing access to safe, healthy, and nutritious food for the growing population.

## Data availability statement

The original contributions presented in the study are included in the article/supplementary material, further inquiries can be directed to the corresponding author/s.

## Ethics statement

Ethical review and approval was not required for the study on human participants, in accordance with the local legislation and institutional requirements.

## Author contributions

SS: validation, resource, and data mining. MP-C: supervision, formal analysis, methodology, and writing—original draft preparation. SG: writing—methodological advice, interpretation, and reviewing and editing. MR: conceptualization, software, and writing—reviewing and editing. All authors contributed to the article and approved the submitted version.

## Conflict of interest

The authors declare that the research was conducted in the absence of any commercial or financial relationships that could be construed as a potential conflict of interest.

## Publisher's note

All claims expressed in this article are solely those of the authors and do not necessarily represent those of their affiliated organizations, or those of the publisher, the editors and the reviewers. Any product that may be evaluated in this article, or claim that may be made by its manufacturer, is not guaranteed or endorsed by the publisher.
